# UK Pigs at the Time of Slaughter: Investigation into the Correlation of Infection with PRRSV and HEV

**DOI:** 10.3390/v9060110

**Published:** 2017-06-09

**Authors:** Jean-Pierre Frossard, Sylvia Grierson, Tanya Cheney, Falko Steinbach, Bhudipa Choudhury, Susanna Williamson

**Affiliations:** 1 Animal and Plant Health Agency, Woodham Lane, New Haw, Surrey KT15 3NB, UK; tcheney87@hotmail.com (T.C.); Falko.Steinbach@apha.gsi.gov.uk (F.S.); Bhudipa.Choudhury@apha.gsi.gov.uk (B.C.); 2 Surveillance Intelligence Unit, Animal and Plant Health Agency, Rougham Hill, Bury St Edmunds, Suffolk IP33 2RX, UK; Susanna.Williamson@apha.gsi.gov.uk

**Keywords:** porcine viruses, PRRSV, HEV, evolution and molecular epidemiology, co-infections

## Abstract

Hepatitis E virus (HEV) and porcine reproductive and respiratory syndrome virus (PRRSV) and are both globally prevalent in the pig population. While HEV does not cause clinical disease in pigs, its zoonotic potential has raised concerns in the food safety sector. PRRS has become endemic in the United Kingdom (UK) since its introduction in 1991, and continues to cause considerable economic losses to the swine industry. A better understanding of the current prevalence and diversity of PRRSV and HEV in the UK, and their potential association, is needed to assess risks and target control measures appropriately. This study used plasma, tonsil, and cecal content samples previously collected from pigs in 14 abattoirs in England and Northern Ireland to study the prevalence of several pathogens including PRRSV and HEV. The diversity of PRRSV strains detected in these samples was analyzed by sequencing open reading frame 5 (*ORF5*), revealing no substantial difference in PRRSV strains from these clinically unaffected pigs relative to those from clinical cases of disease in the UK. Despite the potential immuno-modulatory effect of PRRSV infection, previously demonstrated to affect *Salmonella* and HEV shedding profiles, no significant association was found between positive PRRSV status and positive HEV status.

## 1. Introduction

Hepatitis E virus (HEV) is the cause of hepatitis E in humans, typically a self-limiting hepatitis but more serious in those with pre-existing liver conditions and in the immunocompromised [1]. In pigs, HEV infection alone does not cause clinical disease. HEV genotypes HEV-3 and HEV-4 are the cause of sporadic cases of hepatitis E in developed countries, and are ubiquitous in the pig population worldwide [2]. Hepatitis E is a foodborne zoonosis, for which pork or pork products from infected pigs is one of the risks identified in Europe [1,2] and consumption of processed pork products in the United Kingdom (UK) has been shown to be associated with an increased risk of acquiring HEV [3]. Hence, there is a need to better understand factors influencing HEV entering the food chain.

Porcine reproductive and respiratory syndrome virus (PRRSV) was first confirmed in the UK in 1991 and is now considered endemic [4]. The economic and welfare impacts of the disease are considerable, as both the breeder and grower segments of the pig industry are affected [5]. All PRRSV infections in the UK characterized to date have been identified as being caused by genotype 1 virus, but the genetic diversity of the virus is continually increasing [6]. The phylogenetic analyses of UK PRRSV sequences have previously been based on data from samples submitted for diagnostic purposes, originating from clinical cases of PRRS, thereby possibly introducing a bias in our coverage of circulating strains. It is therefore possible that PRRSV strains circulating in apparently healthy pigs in the UK may represent a different subset from those causing disease.

PRRSV infection has been suggested to modulate pig immune responses, thereby rendering pigs more susceptible to other infections [7,8,9]. For example, previous studies have shown significant associations between PRRSV presence and *Salmonella* shedding [10]. Salines et al. [11] reported that experimental co-infection of HEV and PRRSV affected the dynamics of HEV infection. However, a direct immune-regulation in infected pigs could not be confirmed for genotype 1 PRRSV [12], rather suggesting a role for co-infection viruses influencing each other more directly. Moreover, less pathogenic strains of genotype 1 PRRSV seem to cause a more persistent infection than highly pathogenic ones which are better resolved by the immune response [13]. Notably, viruses closely related to the modified-live vaccine used in the UK have previously been found circulating on farms with clinical PRRS [6].

In 2013, an abattoir-based study was undertaken to estimate the prevalence of various pathogens including HEV and PRRSV in UK-reared pigs at slaughter and seroprevalence for PRRSV was 58.3% (362/621) [14], while HEV seroprevalence was 92.8% (584/629). Approximately 5.7% of pigs were HEV viremic (36/629) and around one in five pigs had evidence of an active HEV infection (129/629), with HEV RNA detected in serum or cecal contents [15]. To follow up these studies, we report here on (1) the investigation of PRRSV active infection (RNA in tonsil) using the same 2013 abattoir survey sample-set and (2) an analysis of the correlation of PRRSV and HEV infection in these pigs, which could be of significance for the control of both diseases and in informing farming practices for reducing HEV in the food chain.

## 2. Materials and Methods

The overall study design for the abattoir survey has been described previously [14]. Briefly, 626 qualifying pigs were sampled at 14 abattoirs in England and Northern Ireland, between January and May 2013. These pigs originated from 439 farms, with between 1 and 10 pigs from each. The majority of pigs were from farms in England (81.7%), followed by Northern Ireland (13.4%), Scotland (4.5%), and Wales (0.3%), which is representative of the UK pig population [16]. Of the samples collected from each selected carcass along the processing line, those relevant for the investigation of HEV and PRRSV were a blood sample (whole blood with ethylenediaminetetraacetic acid (EDTA)), tonsil, and the whole cecum.

Antibody to PRRSV had been detected in 362 of 621 plasma samples, with 11 additional samples being inconclusive [14]. Only 610 of these plasma samples were also analyzed for the presence of antibodies to HEV, with 354 of these being sero-positive for PRRSV. Tonsil samples from all seropositive or inconclusive pigs were then tested by a real-time reverse transcription polymerase chain reaction (RT-PCR) to detect PRRSV nucleic acid. The tonsils of four other pigs for which plasma samples—and hence enzyme-linked immunosorbent assay (ELISA) results—were missing were also tested by PCR, while for five seropositive animals the tonsil samples were not available, so a total of 372 tonsil samples were analyzed; only 358 of these animals also had matching HEV PCR data. RNA was extracted from the tonsil samples using the MagNA Pure LC DNA Isolation kit II (Tissue) following the manufacturer’s instructions (Roche Diagnostics, Burgess Hill, UK), setting the sample volume to 110 μL and the elution volume to 200 μL. A real-time RT-PCR assay targeting the nucleocapsid (*ORF7*) gene of PRRSV and differentiating genotypes 1 and 2 was used [17] with a Stratagene Mx3000P QPCR System (Agilent Genomics, Wokingham, UK). Sequencing of the PRRSV open reading frame 5 (*ORF5*) gene was subsequently performed on nucleic acids from tonsil samples testing positive in the diagnostic PRRSV PCR to characterize the viruses present [6]. The PCR amplicons were purified using the Beckman AMPure solid phase reversible immobilisation (SPRI) technique (Beckman Coulter Ltd., High Wycombe, UK). Cycle sequencing was performed using forward and reverse primers and ABI BigDye chemistry (Applied Biosystems Ltd., Warrington, UK) at the end of which the dye terminators were removed using Beckman CleanSEQ SPRI (Beckman Coulter Ltd., High Wycombe, UK). Samples were sequenced on an ABI capillary electrophoresis DNA analyser (Applied Biosystems Ltd., Warrington, UK) and the raw data analysed by ABI SeqScape software (Applied Biosystems Ltd., Warrington, UK). The resulting sequences were aligned with 48 reference sequences from GenBank, and with 431 other UK PRRSV *ORF5* sequences from samples submitted to the Animal and Plant Health Agency (APHA) for PRRS diagnostic testing between 1991 and 2014, using the ClustalW algorithm [18] in MEGA6 [19]. The phylogenetic tree was generated in MEGA6 using the neighbour-joining method [20], with the evolutionary distances being computed using the maximum composite likelihood method [21]. The sequences obtained were deposited in GenBank with accession numbers MF043094 to MF043116.

Existing data for PRRSV seropositivity [14] and HEV seropositivity and active infection [15] were collated with data obtained in this study for PRRSV active infection. Associations between the two viruses, or antibodies to them, in the same carcasses were investigated using χ^2^ tests, with Stata v.12 (StataCorp, College Station, TX, USA). Collated data for PRRSV seropositivity and HEV seropositivity and active infection (RNA in plasma and/ or cecal contents) were available for 610 pigs. Collated data for PRRSV active infection (RNA in tonsil) and seropositivity and active infection for HEV were available for 358 pigs.

## 3. Results

### 3.1. Detection of Active PRRSV Infection at Slaughter Age

PRRSV RNA was detected in 31 of 372 tonsil samples. This corresponds to 8.3% of the PRRSV seropositive finisher pigs showing active PRRSV infection at slaughter. Importantly, all PCR-positive samples were of genotype 1 (European), which is endemic in the UK, and no genotype 2 virus, which is exotic to the UK, was detected. While the ELISA-positive pigs tested by PCR originated from farms in 24 counties, PCR-positive pigs were only identified from farms in eight of those counties. Almost two-thirds of the PCR-positive pigs were from farms in East Anglia or East Riding and North Lincolnshire. While seropositivity had been found to vary significantly between age groups (*p* = 0.002) with the highest level found in pigs aged less than six months (68.5%) and lowest in those aged >12 months (32.1%) [14], the prevalence of PRRSV RNA-positive tonsils was similar across the age groups (Table 1).

### 3.2. PRRSV Genetic Characterization

Sequencing of the *ORF5* PRRSV gene was undertaken on 29 of the 31 tonsil samples from which PRRSV RNA was detected. Two PCR-positive tonsils were not suitable for sequencing as there was insufficient viral nucleic acid in the samples. Six samples did not yield useable sequence data. All of the sequences confirm that the viruses belong to PRRSV genotype 1. Only three of the sequences may be considered to possibly originate from the currently licensed attenuated vaccine, with greater than 99% similarity between the sample and vaccine strain *ORF5* sequences (99.2%, 99.8%, and 100%).

**Figure 1:**
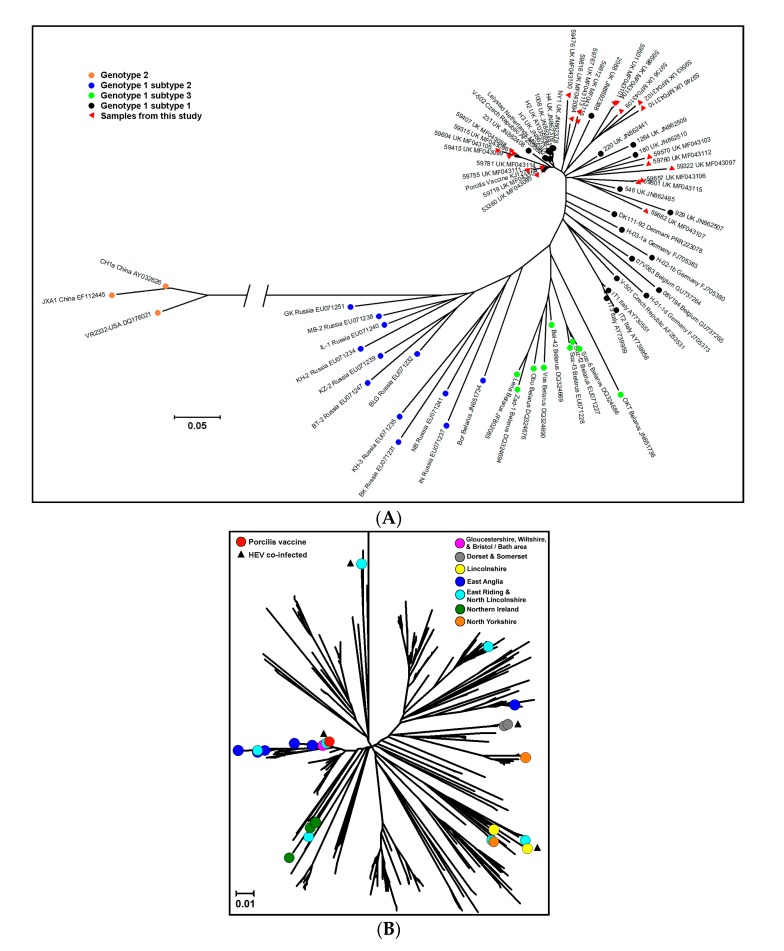
Diversity of porcine reproductive and respiratory syndrome virus (PRRSV) *ORF5* sequences. Alignment of the sequences was performed with the ClustalW algorithm [18], and phylogenetic analyses (neighbour-joining method with bootstrap test) were conducted using MEGA software version 6 [19]. (**A**) Worldwide: The 23 *ORF5* sequences obtained from the polymerase chain reaction (PCR)-positive samples (red triangles) are shown in the context of 48 worldwide reference *ORF5* sequences from different genotypes and subtypes, and the modified-live vaccine sequence. The color of the markers indicates the genotype and subtype of the viruses. The scale bar represents five nucleotide change per 100; (**B**) United Kingdom (UK): The 23 *ORF5* sequences obtained from the PCR-positive samples (colored circles) are shown in the context of 431 other UK *ORF5* sequences dating from 1991 to 2014, and the modified-live vaccine sequence. The black triangles indicate samples that were also positive for hepatitis E virus by PCR. The color of the markers indicates their geographic origin. The scale bar represents 1 nucleotide change per 100.

The phylogenetic trees (Figure 1) illustrate the genetic diversity of the *ORF5* genes from the 23 samples in this study in comparison to the vaccine virus licensed in the UK at the time and 48 published reference sequences representing the different genotypes and subtypes (Figure 1A) and in more detail, in the context of 431 previously sequenced viruses specifically from UK pigs between 1991 and 2014 (unpublished data) (Figure 1B). In the within-UK analysis, there is no clear association between geographic origin and the clade in which the PRRSV strains belong. All of the 23 sequences are found in clades where other UK strains were already identified, and no distinct clustering is observed.

### 3.3. PRRSV and HEV Co-Infections

Analyses were performed to identify potential associations between PRRSV serology or PCR status (PRRSV RNA detected in tonsil sample) and HEV serology or PCR status (HEV RNA detected in serum or cecal contents). These are summarized in Table 2 and Table 3. Of the six animals that were PCR positive for both HEV and PRRSV, one was less than six months old, three were six months of age, and two were greater than six months of age.

There was no evidence from this study that PRRSV infection, as detected by serology or PCR, increased the likelihood of HEV infection in pigs at the time of slaughter. The only associations identified suggested that for the pigs in this study, PRRSV seropositive animals were less likely (17.0% vs. 24.2%, *p* = 0.03) to be HEV PCR positive in plasma or cecum (Table 3); PRRSV seropositive animals were less likely (4.0% vs. 7.8%, *p* = 0.05) to be HEV PCR positive in plasma (Table 2); PRRSV PCR positive animals were less likely (83.9% vs. 95.1%, *p* = 0.01) to be HEV seropositive (Table 3).

## 4. Discussion

It has been suggested that infection with PRRSV renders pigs more susceptible to secondary bacterial [7,8,10,22] or viral [9,23] infections. In this study, we further investigated the active PRRSV infection at slaughter age and the association between PRRSV infection and an active HEV infection in pigs entering the food chain in the UK. Since the majority of UK pig farms that vaccinate against PRRSV use a live vaccine [24] it was considered that PRRSV in both the vaccinated and naturally infected pigs may modulate HEV infection.

The 2013 abattoir survey had found a prevalence of antibodies to PRRSV in slaughter-age pigs of 58.3% [14]. Antibody to vaccine and field PRRSV cannot be distinguished but vaccination of rearing pigs is less common than that of breeding pigs in the UK and is generally performed when there is an expectation of field PRRSV challenge during the rearing period. Therefore, seropositivity in finishers is considered a reasonable indicator of the presence of PRRSV infection on the respective rearing units. From the PRRSV seropositive pigs in the study, the prevalence of PRRS viral RNA in tonsils, where PRRSV may persist up to 130 days post infection [25], was 8.3%. As tonsils from most seronegative pigs were not tested by PCR, it is possible that detection of a few PRRSV-positive pigs in the early stages of infection were missed, although PRRSV RNA was not detected in any of the 28 tonsils from seronegative pigs in a pilot study (data not shown). As the pigs were not showing obvious clinical signs, this is a significant finding, highlighting that a proportion of healthy pigs from PRRSV-infected units may be infectious at slaughter, and if still shedding virus, may be able to transmit the virus on to other farms, for example through contaminated vehicles [26], underlining the need for good biosecurity during and after transport to slaughter. These findings triggered the genetic characterization of the viruses in order to further evaluate the potential risks associated with their presence.

Overall, the sequences from the abattoir samples did not cluster separately from those from clinical cases submitted for diagnostic testing, and they appear to be representative of the overall diversity of PRRSV strains circulating in the UK. None of them was from an Eastern European subtype of genotype 1 PRRSV. Of the 23 successfully-sequenced viruses, three showed greater than 98.5% similarity to the modified-live vaccine used in the UK at the time. These ‘vaccine-like’ viruses may derive from vaccine virus, and have been identified in previous years, although at a lower rate [6], possibly because most samples previously sequenced were from disease outbreaks, whereas these were detected in clinically normal pigs. The other 20 viruses showed between 88.4% and 98.3% similarity to the vaccine sequence. Although the degree of genetic difference of a field PRRS virus from the vaccine strain cannot alone predict the degree of protection that would be afforded by the vaccine to infection by the field virus, nor allow for a determination of the strain’s pathogenicity, these results further illustrate the diverse nature of field PRRSV in the UK. Interestingly, some of the viruses from this study are sufficiently similar to one another to be potentially linked epidemiologically, even when they originate from pigs from different geographic regions. Conversely, for two pigs from the same farm, the viruses detected in them did not show a great degree of similarity. There was no relation between four PRRSV sequences from animals co-infected with HEV (no sequence data was available for the other two), as they each grouped into different clusters in the phylogenetic analysis, one being homologous with the Porcilis PRRS vaccine strain sequence. The sequences from the abattoir samples did not cluster separately from those from clinical cases submitted for diagnostic testing, and they appear to fit within the overall diversity of PRRSV strains previously found to be circulating in the UK. The existence of infected slaughter-age pigs with the potential, if shedding, to transmit the virus to susceptible pigs is thereby confirmed.

We found no evidence in this study that exposure to, or infection with PRRSV enhanced HEV infection rates in pigs entering the food chain. Indeed, pigs exposed to PRRSV were less likely to be viremic (*p* = 0.05). The age-range of the six co-infected pigs identified reflected that of the overall population sampled. The calculated virus load in plasma for HEV showed no association with either PRRSV serology or PCR status (data not shown). Several other studies have specifically investigated PRRSV and HEV co-infection [11,27,28], some by experimental infection. One report of an association of co-infection with disease was restricted to investigation of a single pig [27]. Martelli et al. [28] found no association between these pathogens in an investigation of diagnostic submissions. The 2013 abattoir survey had investigated the presence of a number of other pathogens in clinically healthy pigs, but no evidence was found that the PRRSV-seropositive pigs were more likely to carry *Salmonella* or *Yersinia* or have antibodies to Toxoplasma [14]. Interestingly, Salines et al. [11] reported that experimental PRRSV and HEV co-infection did affect the HEV infection dynamics in five-week old pigs with no maternal antibody for these two endemic viruses. The simultaneous co-infection resulted in delays to both the latent period (13.4 vs. 7.1 days with HEV alone) and the humoral response (43.1 vs. 26.3 days) to HEV, as well as increasing the infectious period (48.6 vs. 9.7 days), in association with an increased HEV viral load. In contrast, several other studies have failed to demonstrate any association between PRRSV infection and the dynamics of other viral infections [13,28,29]. While the present study failed to show any positive association overall between PRRSV and HEV infections, this may be an age-dependent effect, and variation in the timing of the respective infections may also affect their outcomes. Future investigations of natural PRRSV and HEV co-infections should perhaps be directed towards younger pigs, since these were under-represented in this study, and may provide different outcomes. A field study showed that the majority of pigs were infected by 15 weeks of age [30]. Experimental infections to further characterize PRRSV and HEV co-infections must consider non-simultaneous infections, as they may be more relevant to the situation in the field. Other viral co-infections such as porcine circovirus type 2 (PCV-2) and PRRSV or HEV also remain to be investigated, with at least one report of fatal disease associated with HEV and PCV-2 [31].

An active HEV infection in pigs entering the food chain is a potential risk to public health and there is a need to better understand factors that may influence this. The UK abattoir survey had found that one in five pigs had an active HEV infection as they entered the food chain [15]. This same finding of active HEV infections in slaughter-age pigs is found worldwide [32,33,34,35]. There was no evidence from the current study that PRRSV infection adversely affected the proportion of HEV infected pigs entering the food chain. Further studies are needed to investigate factors influencing the dynamics of HEV infection in the pig and within farms and that may then be used to inform means of reducing infection in slaughter-age pigs.

## 5. Conclusions

No association was found between PRRSV and HEV infections in the slaughter age pigs sampled. In addition, there was no difference in strain diversity of PRRSV sampled from clinically unaffected pigs in this study relative to those identified from clinical cases of disease in the UK.

## Figures and Tables

**Table 1:** Porcine reproductive and respiratory syndrome virus (PRRSV) polymerase chain reaction (PCR) results for 372 tonsil samples from PRRS seropositive or inconclusive animals, by age.

Age	Number Tested	Number Positive	% Positive
<6 months	34	4	11.8%
6–12 months	312	25	8.0%
>12 months	19	2	10.5%
Not known	7	0	0%

**Table 2:** Association of hepatitis E virus (HEV) with porcine reproductive and respiratory syndrome virus (PRRSV) seropositivity (from 610 plasma samples where both tests were performed).

Pathogen	Serology Status	HEV			
		Seropositive *n* (%)	RNA + Plasma and/or Cecum *n* (%)	RNA + Plasma *n* (%)	RNA + Cecum *n* (%)
PRRSV	Seronegative (*n* = 256)	235 (91.8)	62 (24.2)	20 (7.8)	50 (19.5)
	Seropositive (*n* = 354)	333 (94.1)	60 (17.0)	14 (4.0)	52 (14.7)
		*p* = 0.31	*p* = 0.03	*p* = 0.05	*p* = 0.13

**Table 3:** Association with porcine reproductive and respiratory syndrome virus (PRRSV) active infection (RNA detection in tonsil) (from 358 animals where PCR results for both PRRSV and hepatitis E virus (HEV) were available).

Pathogen	RNA Status	HEV			
		Seropositive *n* (%)	RNA + Plasma and/or Cecum *n* (%)	RNA + Plasma *n* (%)	RNA + Cecum *n* (%)
PRRSV	PCR negative (*n* = 327)	311 (95.1)	55 (16.8)	12 (3.7)	48 (14.7)
	PCR positive (*n* = 31)	26 (83.9)	6 (19.4)	2 (6.5)	5 (16.1)
		*p* = 0.01	*p* = 0.73	*p* = 0.45	*p* = 0.83
